# Synthesis of BODIPY@MOFs as Hybrid Materials for Emerging Applications: A Review

**DOI:** 10.3390/molecules30244790

**Published:** 2025-12-16

**Authors:** Louise Bureller, Clément Michelin, Federico Cisnetti

**Affiliations:** Université Clermont Auvergne, Clermont Auvergne INP, CNRS, ICCF, 63000 Clermont-Ferrand, France; louise.bureller@uca.fr

**Keywords:** BODIPY, Metal–Organic Frameworks, photodynamic therapy, photocatalysis, luminescent materials, sensing

## Abstract

This review explores the integration of Boron-Dipyrromethene (BODIPY) dyes within Metal–Organic Frameworks (MOFs), highlighting their combined potential in various applications. MOFs, with their high porosity and structural versatility, provide an ideal platform to enable applications with BODIPYs, which otherwise remain challenging in the solid state. The article discusses different strategies for incorporating BODIPYs into MOFs, including their use as monodentate, bidentate, and tridentate ligands, as well as covalent attachment and non-coordinating encapsulation. The resulting hybrid materials exhibit enhanced properties suitable for applications in the luminescent materials/light harvesting, photodynamic therapy, sensing, and photocatalysis areas. The review emphasizes the importance of synthetic conditions, characterization techniques, and the quantification of BODIPY loading to ensure the integrity and functionality of the MOF structures.

## 1. Introduction

This short comprehensive review of the literature published up to October 2025 deals with the interplay between two hot research areas, BODIPYs as exquisite molecular fluorophores and Metal–Organic Frameworks (MOFs) as host materials. Before discussing the complete series of examples from this specific but rapidly expanding research area, we will first introduce BODIPYs and their applications that could benefit from a hybrid materials approach and then MOFs. This topic has only been covered partially in previous reviews [1,2,3].

In this review, we will shortly introduce both BODIPYs and MOFs, before reviewing the literature with an emphasis on synthetic conditions and materials characterization (with the scope of both verifying and quantifying the inclusion of BODIPY *and* the integrity of the MOF). The applications will also be briefly discussed and used to organize the examples thematically.

**Figure 1:**
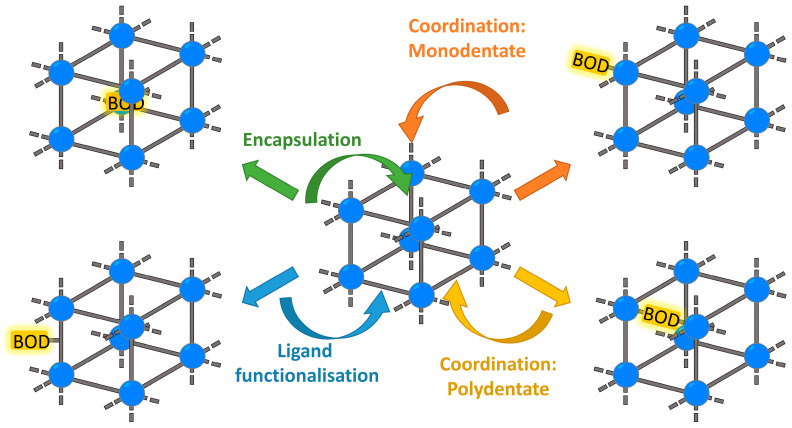
Overview of synthetic strategies for BODIPY@MOF materials. Lines: organic linkers. Balls: metallic nodes.

Four different approaches will be discussed in this review: monodentate coordination (Section 2.1), polydentate coordination (Section 2.2 and Section 2.3), covalent ligand functionalization (Section 3), and encapsulation (Section 4). They are depicted in Figure 1.

### 1.1. BODIPYs and Their Applications

**Figure 2:**
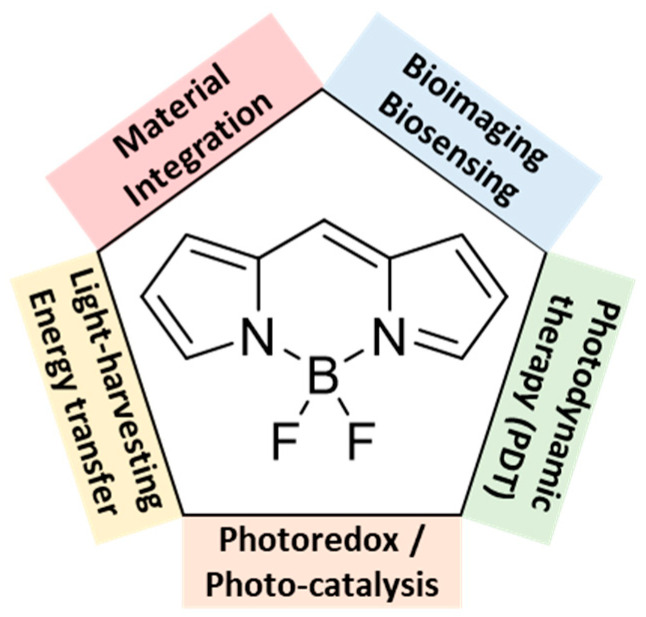
Main application of BODIPY dyes.

Boron-dipyrromethene (BODIPY) dyes constitute one of the most widely studied families of fluorophores owing to their outstanding photophysical and chemical properties. They typically exhibit intense absorption bands, narrow emission profiles with high fluorescence quantum yields *in solution*. They are usually associated with small Stokes shifts, which might be a limitation for some applications. In addition, BODIPY derivatives exhibit remarkable stability and tolerate a wide range of chemical environments, a property that largely accounts for their successful use in biological systems [4]. The wide range of applications of BODIPY dyes, spanning from bioimaging to photonic and hybrid materials, is illustrated in Figure 2.

**Figure 3:**
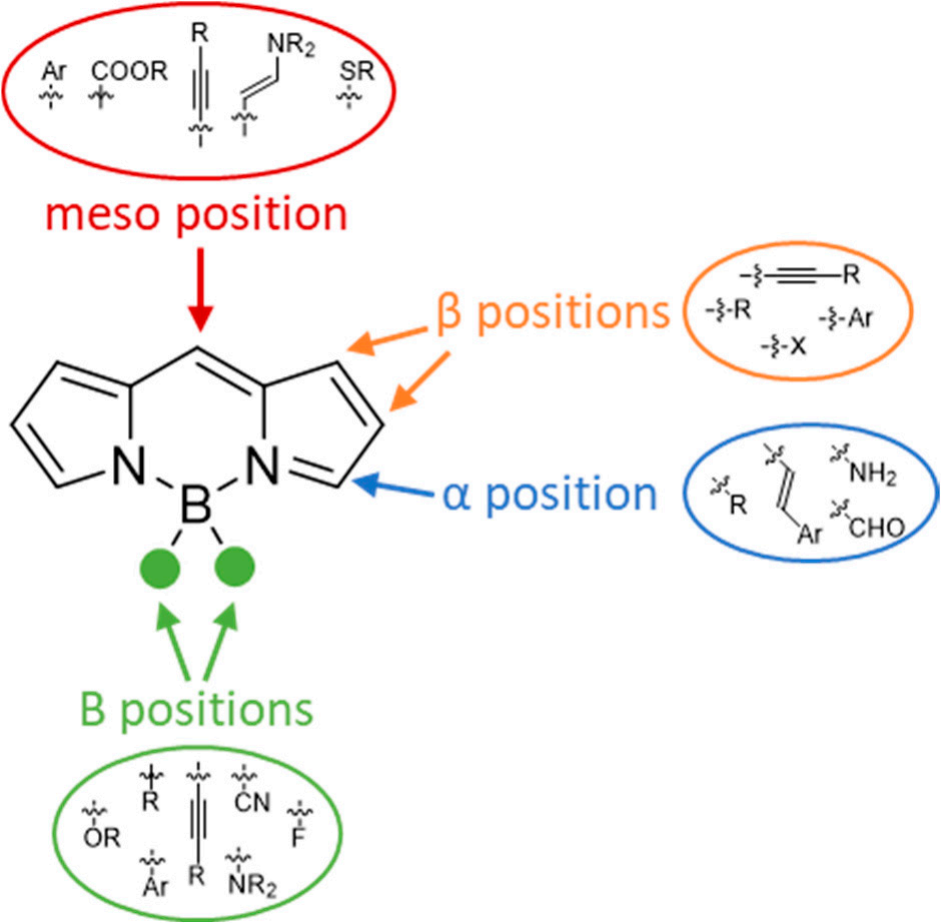
Structure of the BODIPY core highlighting the main substitution positions (meso, α-, β-, and boron sites) commonly used to tune photophysical and chemical properties.

A key advantage of BODIPY dyes lies in their remarkable structural tunability. The dipyrromethene core can be decorated at multiple positions with a wide range of substituents, providing straightforward access to derivatives with tailored properties. The most common substitution sites and typical functionalization patterns of BODIPY dyes are illustrated in Figure 3.

Substitution at the meso position is commonly used to introduce functional handles for conjugation. Substituents at the α- and β-positions can modulate electronic properties, control photophysical behaviour, and extend conjugation, enabling bathochromic shifts in the spectra [5]. Furthermore, functionalization at the boron centre itself allows additional modulation of stability and solubility. This high degree of modularity makes it possible to design BODIPY dyes with customized optical properties [6].

Despite these advantages, BODIPY dyes, like many organic fluorophores, suffer from a significant drawback for solid-state applications; their fluorescence is often quenched in the solid state due to aggregation-caused quenching (ACQ). Strong π–π stacking interactions between BODIPY chromophores can lead to non-radiative deactivation pathways, resulting in quenched fluorescence at high concentrations or in condensed phases. This limitation restricts their direct use in solid-state optical materials and has motivated the development of strategies to suppress ACQ, such as chemical modification, aggregation-induced emission (AIE) design, or incorporation into host matrices [7,8].

Through appropriate decoration and functionalization, BODIPY dyes have been adapted for a wide spectrum of applications. They have found extensive use in bioimaging and chemical sensing, where their sharp emission profiles and high brightness are particularly advantageous. Their ability to generate reactive oxygen species has also made them attractive candidates for photodynamic therapy (PDT), especially for halogenated BODIPYs [9]. In addition, BODIPYs are increasingly explored as efficient light-harvesting antennas in energy transfer processes and as active components in photocatalysis and optoelectronic devices [10,11,12,13]. Most importantly, the introduction of functional groups such as carboxylates or pyridyl substituents provides anchoring sites that can be exploited for integration into hybrid materials, including coordination polymers and metal–organic frameworks (MOFs).

**Scheme 1:**
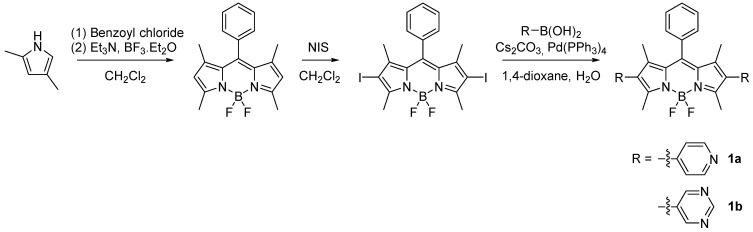
Classical BODIPY synthesis and common β-functionalisation pathways, exemplified for BODIPYs **1a** and **1b**.

In this context, the association of BODIPY dyes with metal–organic frameworks (MOFs) has emerged as a powerful strategy to overcome the intrinsic limitations of these fluorophores in the solid state. MOFs provide well-defined porous architectures (see next subsection) that spatially isolate the chromophores, thereby suppressing aggregation-caused quenching and preserving the high fluorescence efficiency of BODIPYs. Their tunable chemical environment also enhances the stability of the dyes and enables fine control over their photophysical and photochemical behaviour. Furthermore, the easy introduction of anchoring (coordinating) groups on BODIPY derivatives facilitates their integration into MOF structures, giving rise to hybrid materials that combine the optical performance of BODIPYs with the versatility of MOFs for sensing, photocatalysis, and light-harvesting applications. The BODIPY chromophores discussed in this review are, for the most part, synthesized using the classical route to BODIPY dyes, which involves constructing a dipyrromethene intermediate followed by BF_2_ complexation [6]. Owing to its reliability and functional-group tolerance, this method remains the dominant approach for accessing the derivatives later incorporated into MOF systems. β-Functionalisation is most commonly achieved through halogenation (Br or I), providing reactive sites for diversification via cross-coupling reactions such as Suzuki–Miyaura or Sonogashira couplings. An example of this synthetic pathway for one of the most used BODIPY-derived ligands [14] is shown in Scheme 1.

Finally, α-methyl groups can be functionalized through Knoevenagel-type condensations, extending the π-conjugation and inducing a bathochromic shift in the absorption and emission wavelengths [5].

### 1.2. Metal–Organic Frameworks: General Considerations and Applications

Discovered in the late 20th century, Metal–Organic Frameworks (MOFs) are hybrid materials combining organic and inorganic components. They consist of metal nodes (metal ions or clusters) acting as Lewis acids, and organic ligands that are at least bidentate and non-chelating, considered as Lewis bases [15]. MOFs are thus three-dimensional coordination polymers based on metal-ligand bonds and exhibit well-defined crystalline structures. These materials are distinguished by their high porosity, which allows them to capture various molecules such as solvents, gases, pollutants, etc. [16]. The pioneering work in this field has very recently earned Richard Robson, Susumu Kitagawa, and Omar Yaghi the 2025 Nobel Prize for the development of these innovative materials. While an exhaustive introduction of the field of MOFs (more than 65,000 references as of October 2025) is completely outside the scope of this review, we will focus herein on some of their core characteristics, as well as their applications to illustrate the possible synergies with the BODIPY guests described in the following sections.

MOFs come in several families with various common names. In some cases, their names refer to the institution where they were discovered, such as the University of Oslo (UiO) or *Matériaux de l’Institut Lavoisier* (MIL). Others; however, provide insights into their structure. This is particularly the case for the Zeolitic Imidazolate Framework (ZIF) family, whose three-dimensional network is topologically very similar to that of zeolites. These materials have metal nodes composed of a tetrahedral metal and imidazolate ligands. The family of Porous Coordination Networks (PCN) is a porous network with a stereo-octahedron geometry, often associated with porphyrin-centred ligands; PCN-222 is a representative example [17]. Porous Coordination Polymers (PCPs) also belong to the large family of MOFs. They are distinguished by the use of carboxylate and pyridinic ligands associated with transition metals. Finally, another particular class is that of Isoreticular Metal–Organic Frameworks (IRMOF), which are isoreticular to MOF-5, also known as IRMOF-1. These MOFs use aromatic ligands, such as terephthalic acid, and present similar topologies, differing mainly by the substitutions on the aromatic ring [18].

**Figure 4:**
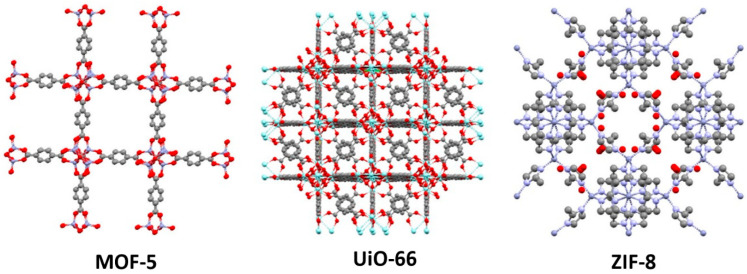
Crystal structures of archetypical MOFs (CCDC numbers): MOF-5 (256965) [22], UiO-66 (733458) [20], and ZIF-8 (602542) [21]. Figure generated with Mercury software 4.0 [23].

Among the many listed MOFs, MOF-5, UiO-66, and ZIF-8 (Figure 4) are some of the most frequently studied. We will detail here some elements of their structure as representative examples. MOF-5, with the formula Zn_4_O(C_8_H_6_O_4_)_3_, consists of tetrahedral zinc clusters {Zn_4_O}^6+^ connected by terephthalate ligands. Its crystalline structure forms an extended three-dimensional cubic network. This MOF was reported by O. Yaghi’s group in 1999 [19]. UiO-66, synthesized by K. Lillerud in 2008 at the University of Oslo, has the formula Zr_6_O_4_(OH)_4_(C_8_H_4_O_4_)_6_ [20]. It is composed of zirconium metal clusters (Zr^4+^) connected by terephthalate ligands. Its crystalline structure exhibits a face-centred cubic (fcc) geometry. UiO-66 is renowned for its robustness, particularly its resistance to various solvents such as water (except at extreme pH), DMF, or acetone. Finally, ZIF-8 (Figure 4), discovered in 2006 by O. Yaghi, with the formula Zn(C_4_H_6_N_2_)_2_, consists of Zn^2+^ ions connected by 2-methylimidazole ligands. It is archetypal of the ZIF family (see above) [21]. This material is distinguished by its stability in water and at high temperatures (550 °C), and also presents good chemical stability.

MOFs generally have relatively harsh synthesis methods compared to usual organic synthesis. Indeed, they are often obtained by solvothermal synthesis, a technique performed at high temperature and pressure in sealed autoclaves [24]. The temperature used is generally above the boiling point of the solvent with added pressure, which allows the dissolution of all precursors (even the most difficult to dissolve), and provides crystallization conditions similar to those governing the geological formation of minerals. However, some MOFs can also be obtained by slow vapour diffusion. This technique consists of dissolving the reagents in a solvent A, and then placing this solution in a closed container together with solvent B, in which the solubility of the product is reduced. Examples for MOF-5 (in this particular case, the vapour diffusion brings a weak base to the reaction medium), UiO-66, and ZIF-8 are presented in Table 1. These processes improve the crystallinity of the MOF, and solvothermally synthesized MOFs are generally more crystalline than those obtained under milder conditions [25]. However, hydrothermal conditions might be challenging for organic fluorophore guests such as BODIPYs. Moreover, when organic linkers of the MOF consist of carboxylates and are introduced in the synthesis in their protonated state, the synthetic conditions imply Brønsted acidity in addition to the Lewis acidity of the metal precursors. Both of them can prove challenging to BODIPYs. However, alternative milder methods are now being studied to avoid the harsh solvothermal conditions. For example, MOF-5 can be synthesized at room temperature in the presence of a weak base, triethylamine, according to a method published by Yaghi [26]. Other synthesis processes in aqueous media are also now documented [27].

MOFs are known for their numerous applications, particularly in gas or solvent capture. Due to their exceptionally high porosity, they are capable of selectively or non-selectively capturing various gases, thus enabling gas separation. Additionally, these materials can act as solvent traps, generally by occupying the cavities or channels of the porous structure [29,30]. Another frequently studied application is catalysis, and more specifically, photocatalysis. Some MOFs, due to their photochemical properties, can thus be used as photocatalysts [31,32]. In some cases, MOFs also exhibit interesting luminescence properties for detection applications [33,34]. This luminescence can originate from the ligand, if it is a fluorophore, or from the metal if it has emissive properties. Charge transfer from the metal to the ligand or vice versa can also induce material emission [35,36]. The interested reader is referred to further specialized reviews (e.g., [37,38,39,40,41,42]).

## 2. BODIPYs as Ligands

This section focuses on BODIPY@MOF materials, where BODIPY has been modified to confer coordinating properties. The examples will be organized by increasing denticity of the BODIPY ligand.

### 2.1. Monodentate Ligands

**Scheme 2:**
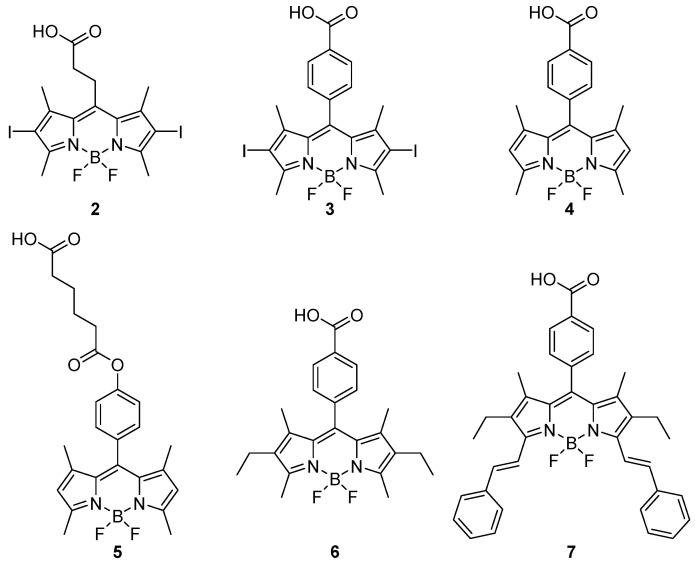
Monodentate BODIPYs **2**–**7**.

In order to incorporate BODIPY into MOF structures, some groups have chosen to functionalize BODIPY by adding a single coordinating arm (BODIPY **2**–**7** depicted in Scheme 2). Synthetically, the meso position is the most easily modifiable. This approach allows the fluorophore to be integrated as a monodentate ligand within the structure for various applications. However, from a thermodynamic perspective, the substitution within the MOF of polydentate ligands with monodentate ones is highly unfavourable. To overcome this hurdle, coordination to additional node sites—i.e., those not occupied by the MOF linkers—or to sites resulting from defects can be considered. For this subsection, synthetic strategies and results in terms of BODIPY incorporation (specifically important in this case as no defined stoichiometry is expected) are summarized in Table 2. The synthetic conditions highlight that, whatever the incorporation procedure, mild conditions compatible with the BODIPY chromophore were used, a distinctive feature that differs markedly from standard MOF synthesis conditions (typically, high-temperature solvothermal).

In many cases, these applications relate to the **biomedical field**, such as photodynamic therapy (PDT) or the development of antibacterial systems. For instance, Xie et al. reported in 2016 the formation of a material capable of acting as an anticancer agent [43]. In this study, BODIPY **2** was functionalized with a 2-carboxyethyl group at the meso position and two iodine atoms at the β positions. This BODIPY was immobilized within a nanoscale UiO-66-type MOF, which has the formula Zr_6_O_4_(OH)_4_(C_8_H_4_O_4_)_6_ and features Zr^4+^ metal clusters connected by terephthalate linkers [20]. BODIPY **2** was incorporated into the structure using the solvent-assisted ligand exchange (SALE) method—a post-synthetic approach allowing the exchange of one ligand for another. In this case, the ditopic terephthalate ligand was partially replaced by the monotopic BODIPY. After the SALE procedure, the material was washed multiple times with (DMF, MeOH) to remove any BODIPY molecules trapped within the pores. Incorporation was confirmed by transmission electron microscopy (TEM) with elemental mapping of iodine, revealing a relatively homogeneous distribution. Additionally, the decrease in specific surface area from 1591 m^2^/g to 1422 m^2^/g after SALE is consistent with successful incorporation. The appearance of two characteristic signals of **2** in the IR and solid-state ^1^H NMR spectra. Inductively coupled plasma mass spectrometry (ICP-MS) and UV-Vis analysis of the washing solutions allowed quantification of the BODIPY within the structure. Powder X-ray diffraction (PXRD) analyses showed that this modification had minimal impact on the MOFs’ crystalline structure. Biological tests demonstrated that the material exhibits good biocompatibility in the dark, significant photosensitivity and cellular uptake, and a notable ability to generate singlet oxygen (^1^O_2_). Under irradiation, **2**@UiO-66 effectively reduced the viability of B16F10, CT26, and C26 cancer cells.

In 2019, Gupta et al. reported BODIPY **3** functionalized with a carboxylate group at the meso position and iodine atoms at the β positions [44]. This derivative was incorporated via solvent-assisted ligand incorporation (SALI). Unlike the previously mentioned SALE approach, **3** was directly coordinated to the preformed MOF cluster in nanoscale PCN-222 [17], a material with the formula Zr_6_O_8_(OH)_8_(C_48_H_30_N_4_O_8_)_2_, where Zr^4+^-containing metal clusters are connected by tetradentate porphyrin linkers. The material was washed (acetone/methanol, then acetone) to remove any uncoordinated BODIPY, confirming that the remaining **3** in the MOF was complexed. Again, the specific surface area decreased in the presence of BODIPY, from 1755 m^2^/g to 1015 m^2^/g. NMR analysis after degradation (D_2_SO_4_ (10%) in DMSO-d_6_) revealed the presence of BODIPY **3** within the structure. Quantitative analysis indicated a ratio per Zr_6_ cluster of 8 porphyrins to 3.5 BODIPYs, corresponding to 44% BODIPY relative to the porphyrins in the structure. Successful integration of BODIPY into the MOF was further confirmed by (the disappearance of the OH stretching of Zr clusters). The resulting material exhibited significant anticancer activity under irradiation, showing 10 to 20 times greater cytotoxicity against MCF-7 and B16F10 cancer cells compared to PCN-222 alone.

Shen, Linhardt et al. described in 2021 a synthesis of ZIF-90 modified with **4**. Despite the low loading, PXRD showed deep structural modifications of the resulting material as compared to pristine ZIF-90, which can be related to the different coordination pattern of **4** as compared to imidazolate ligands. Nevertheless, the resulting material allowed the detection of protamine (arginine-rich protein) and heparin in solution, including in realistic conditions (serum) for the former [45].

In a publication focused on designing an antibacterial material, Qu et al. described in 2022 the use of the same BODIPY **4** functionalized with a carboxylate group at the meso position, which was then integrated as a monodentate ligand into a ZIF-8 MOF [46]. This MOF consists of Zn^2+^ ions connected by 2-methylimidazole (formula: Zn(C_4_H_6_N_2_)_2_) [21], aiming to develop a photosensitive antibacterial material. The synthesis of ZIF-8 was carried out under standard conditions, with BODIPY added after a certain time. Powder X-ray diffraction (PXRD) analysis of the resulting material still matched the diffraction peaks of pristine ZIF-8. SEM images revealed hexagonal particles with a diameter of approximately 350 nm for ZIF-8 and rough, hollow nanospheres of about 300 nm for **4**@ZIF-8; these morphological changes were correlated with the integration of BODIPY into the structure. Energy-dispersive X-ray spectroscopy (EDS) confirmed a homogeneous dispersion of **4** within the MOF structure. IR spectroscopy also highlighted the presence of **4** in the structure through observation of characteristic bands. The optical properties of the material were studied using UV-vis and photoluminescence spectroscopies, revealing an absorption peak centred at 465 nm and subsequent emission. Porosity analysis further demonstrated the integration of BODIPY, as the specific surface area decreased from 1351 m^2^/g for ZIF-8 to 612 m^2^/g for **4**@ZIF-8. This material exhibited notable antibacterial activity, inhibiting the growth of *E. coli* and *S. aureus* after 1.5 h under illumination.

In an approach using **photochemistry for chemical applications**, Farha et al. described in 2017 a material active in photo-oxidation [47]. They integrated a BODIPY **5**, modified with a carboxylate-bearing chain at the meso position, into the MOF NU-1000 [50] using the SALI method. This MOF is described by the formula Zr_6_O_8_(OH)_8_(C_44_H_26_O_8_)_2_ and consists of zirconium clusters connected by 1,3,6,8-tetra(4-carboxylphenyl)pyrene linkers. No extensive characterizations to confirm the insertion of BODIPY into the MOF have been reported. However, the analysis of the specific surface area indicated a significant decrease upon incorporation of **5**, from 2100 m^2^/g to 1100 m^2^/g, suggesting successful integration. This assembly enabled the formation of a photocatalyst capable of detoxifying sulphur mustards.

In studies focusing on the **optical properties of the fluorophore within the material**, other applications have also been explored. For example, Kim et al. in 2020 investigated the Förster Resonance Energy Transfer (FRET) phenomenon between BODIPY and a porphyrin (tetrakis(4-carboxyphenyl)porphyrin, TCPP) [48]. To achieve this, they incorporated BODIPY **4** and its diiodinated analogue **3** into a nanoscale MOF of the nPCN-222 type, which also contains {Zr_6_O_8_} nodes as in the previous example. They obtained nanoparticles in the form of small rods, with varying sizes depending on the composition; the control undoped nPCN-222 nanoparticles measure 72 nm in length, while the **4**@nPCN-222 and **3**@nPCN-222 nanoparticles measure 90 nm and 88 nm, respectively. The BODIPYs were incorporated via post-synthetic modification using the SALI method, with an excess relative to the MOF linker, which did not allow precise control of the amounts actually introduced into the MOF structure. NMR (decomposition in D_2_SO_4_ (10%)/DMSO-d_6_) was used to quantify the BODIPY relative to the porphyrins. The ratios of BODIPY to porphyrins per Zr_6_ cluster were 3.6 and 3.5 for **4**@nPCN-222 and **3**@nPCN-222, respectively. The authors studied the FRET phenomenon using luminescence spectroscopy and showed that the excitation of BODIPY at 500–600 nm could result in the emission of TCPP at 630–750 nm within the material.

In a different approach, Cisnetti et al. reported in 2023 a strategy to limit the ACQ (Aggregation-Caused Quenching) effect by diluting a BODIPY fluorophore in MOF-5 [49]. MOF-5 has the formula Zn_4_O(C_8_H_6_O_4_) and consists of zinc clusters connected by terephthalic acid [19]. To achieve this, the authors synthesized a BODIPY **6** functionalized at the meso position with a carboxylate (phenylene spacer) and attempted to integrate it into MOF-5 during synthesis. A room-temperature and neutralized (NEt_3_) procedure was chosen to ensure compatibility with **6** [26]. The fluorophore was incorporated directly during synthesis, adjusting its percentage relative to the linker. The resulting material was washed several times with chloroform to remove any uncoordinated BODIPY, confirming that the remaining **6** was indeed bound within the structure. The obtained MOF exhibited a pink colour and emitted green fluorescence (525 nm) while irradiated under UV light (365 nm). An additional control test was conducted by replacing **6** with a non-coordinating BODIPY (**23**, see Section 4), which did not yield a luminescent MOF, further confirming the coordination of **6** within the structure rather than mere encapsulation. The sample was characterized by powder X-ray diffraction (PXRD), and the observed crystalline pattern matched the simulated pattern of MOF-5. The specific surface area was also measured, showing a 44% decrease between the control and doped MOF-5 samples, providing further evidence of the incorporation of **6**. A second BODIPY **7**, similar to 6 but functionalized with two aromatic arms at the α positions via Knoevenagel condensation with benzaldehyde, was also used. **6**@MOF-5 exhibited a photoluminescence quantum yield (PLQY) of 45% (external PLQY, accounting for absorption: 27.3%) for its best sample containing 10.6 wt-%. For **7**, the optimal properties (52% PLQY and 16% ePLQY) were obtained for a sample containing 5.2% wt-% of BODIPY **7**. For the sake of comparison, the authors measured the quantum yields of **6** and **7** in the synthetic medium (DMF/Et_3_N) and obtained 54 and 77%, respectively. This highlights that the MOF is allowed to obtain solid-state luminescence properties comparable to solution ones.

### 2.2. Bidentate Ligands: MOFs Containing Exclusively BODIPY Ligands

**Scheme 3:**
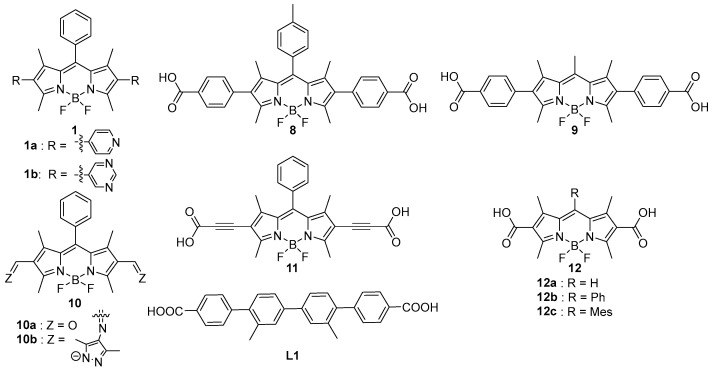
Bidentate BODIPYs **1** and **8**–**11** functionalized and ligand **L1** used along one of these in a single-crystal to single-crystal ligand exchange.

In this section, we will discuss examples of bidentate ligands centred on BODIPYs. All examples involve dual functionalization at the β positions, which ensures an angle close to 180° between the two coordinating functions (Scheme 3). Table 3 overviews the synthetic conditions used for the preparation of MOFs from these ligands. The reaction conditions are slightly harsher than the example discussed in the former subsection, to ensure crystallization. Indeed, in articles discussed within this subsection, the MOF is described by a precise chemical formula (assuming no structural defects), and most frequently, the proof of structure is provided by single-crystal crystallography (SCXRD), coupled with powder X-ray crystallography (PXRD) to prove phase purity.

**Figure 5:**
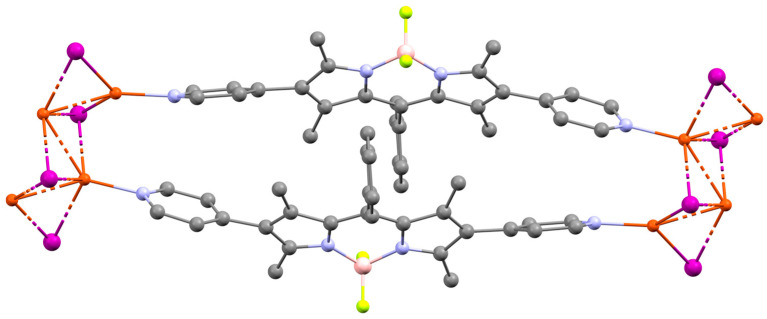
Crystal structure of 1a/Cu_4_I_4_ CCDC number: 1920194 [14]. Figure generated with Mercury software [23].

In a seminal research article focused on the impact of cluster differences on the **structure and crystallinity of MOFs**, Champness et al. used in 2019 two BODIPYs functionalized with two pyridines (**1a**) or two pyrimidines (**1b**) at the β positions [14]. Their reaction in the presence of copper(I) iodide led to the formation of Cu_4_I_4_ clusters in the presence of **1a**, with which they formed one-dimensional chains. However, in the presence of **1b**, the resulting clusters were Cu_2_I_2_, but in this case, alternating stacks of fluorophores and clusters are observed, generating a 3D network. Thus, this study illustrates how modifying the BODIPY functionalization allows control over the topology of the obtained structures. Additionally, photophysical studies were conducted showing the emission of BODIPYs in solution (PLQY of 86% for **1a** and 90% for **1b** in CH_2_Cl_2_ solution) and the loss of this luminescence once integrated into the material. To exemplify a structure typical of MOFs constructed exclusively from BODIPY linkers, the crystal structure of **1a**/Cu_4_I_4_ is depicted in Figure 5.

With this type of ligand, some studies have focused on **applications in photodynamic therapy** (PDT). Meng et al. described in 2024 a BODIPY **8**, substituted at the β position with a carboxylate group, which was then linked to rare earth ions (RE^3+^, RE = Y, La, Ce, Pr, Nd, Eu, Gd, Tb, Er, Tm, Yb, and Lu) to form a set of MOFs [51]. The structures were elucidated using single-crystal X-ray diffraction, revealing a three-dimensional structure where the metal centre has a coordination number of 8, with different phases for lanthanum and cerium on one hand, and for all the other tested ions on the other [52]. The authors thus developed a new PDT material that can be used with different rare earths in vitro on HeLa cancer cells as well as in vivo on tumours caused by these cells in mice. In vivo, **8**@Gd-MOF showed good results in sonophotodynamic therapy (SPDT), reducing tumour volume by 86%. The same Gd-based MOF was used in another study by the same group, in combination with a heterostructure based on NaLnF_4_ nanoparticles capable of upconversion (808 nm → 515–564 nm) via a Nd^3+^ ⟶ Yb^3+^ ⟶ Er^3+^ cascade. The MOF was synthesized in situ on preformed nanoparticles. The overall system resulted in the excitation of BODIPY **8** via near-infrared (NIR) radiation, which has high tissue penetration. In vivo experiments demonstrated that 808 nm NIR light was more effective than visible light to reduce tumour volume in a murine model (90% reduction vs. 65% for 505 nm).

In another recent publication (2024), Fairen–Jimenez et al. first synthesized an isoreticular MOF to UiO-68 named 69-Me_2_ [57] with a ligand **L1**, then proceeded with ligand exchange using a bidentate BODIPY **9**, functionalized at the β positions with paracarboxyphenyl groups [53]. To achieve this, the MOF 69-Me_2_ was incubated with **9**, observing a complete visual change from colourless to red. To verify if all the ligands had been exchanged, they analyzed the degradation products of the MOF using ^1^H NMR, which demonstrated that 100% of the initial ligand had been substituted in what proved to be a single-crystal to single-crystal reaction. The authors then coated their MOF nanoparticles with fluorinated polyethylene glycol and integrated them into a hydrogel based on hydroxyethyl cellulose. This allowed the injection of the MOF containing **9** into a murine tumour. ROS were generated under irradiation by a green LED at 525 nm, leading to a tumour regression of up to 80%.

**Photocatalysis** is another application field that has been studied. Notably, Li et al. synthesized in 2023 a BODIPY **10a** functionalized at the β positions with aldehyde groups and an aromatic cycle at the meso position [54]. Their strategy is original as the actual BODIPY ligand is assembled on preformed clusters via aldehyde-imine condensation. Indeed, **10a** reacted initially with preformed copper(I) clusters Cu_3_L_3_, where L is a pyrazolate bearing a primary amine. Thereafter, during the MOF formation step, the aldehydes of **10a** reacted with the primary amines of the clusters to form covalent imine bonds in **10b**. The resulting MOF was named JNM-20. Its structure was modelled, although single-crystal structures were not reported. It was characterized by PXRD, consistent with the predicted model (albeit with limited crystallinity). NMR and IR analyses were performed to confirm the clear disappearance of the strong C=O bond (1665 cm^−1^) and the formation of the weak C=N bond (1616 cm^−1^). This material serves as a catalyst for synthetic interest. It combines a catalytic copper site with a photocatalytic site (**10b**) to enable the formation of ^1^O_2_. This material promotes the anti-Markovnikov transformation of terminal alkenes or alkynes into primary alcohols.

In 2025, Liu et al. recently published a study investigating the effect of cluster size on MOF structure [55]. A BODIPY **11**, substituted at the β positions with alkynes bearing carboxylic acid groups (obtained via Sonogashira coupling with trimethylsilylacetylene followed by reaction with CO_2_), was reacted with a zirconium salt (ZrOCl_2_.8H_2_O), forming two types of clusters: (Zr_6_(µ_3_-O)_4_(µ_3_-OH)_4_) or (Zr_12_(µ_3_-O)_8_(µ_3_-OH)_8_(µ_2_-OH)_6_). Depending on the cluster size, the unit cell shape differed. For the former cluster, the unit cell was face-centred cubic, while for the latter, it was hexagonal close-packed. The materials were applied in the photocatalytic reduction in CO_2_ to CO. Under UV irradiation, both materials showed promising results with nearly 100% selectivity, yielding CO at 16.72 µmol g^−1^ h^−1^ for **11**@Zr_12_ and 13.91 µmol g^−1^·h^−1^ for **11**@Zr_6_.

In a comprehensive and very recent study (2025), Fairen–Jimenez et al. described various BODIPYs functionalized at the β positions with a carboxylic acid: **12**. These BODIPYs act as ligands towards Zr_6_O_4_(OH)_4_ [56]. The BODIPY **12a**, integrated into a MOF, was then post-functionalized with different groups at the α and free β positions. Knoevenagel-type condensation reactions with aldehydes allowed modification of the chromophore’s absorption, covering a large part of the visible spectrum. Finally, the same BODIPY **12a** was combined with zirconium and other ions such as Sc^3+^, Ti^4+^, V^4+^, and Sn^4+^, leading to bimetallic clusters. The proportion of metal incorporated into each node relative to zirconium was determined by ICP-MS: 83% Sc, 87% Ti, 50% V, and 46% Sn. SEM analyses showed a uniform distribution of the various metals. These different functionalized materials were used in photocatalytic applications on tertiary aniline substrates under ordinary atmosphere: Csp^3^–H carbamoylation in the presence of isonitriles, or dealkylation/acylation in the presence of acyl chlorides or arenesulfonyl chlorides, with very satisfactory yields.

### 2.3. Bidentate Ligands: MOFs Containing BODIPY Ligands and Auxiliary Ligands

**Scheme 4:**
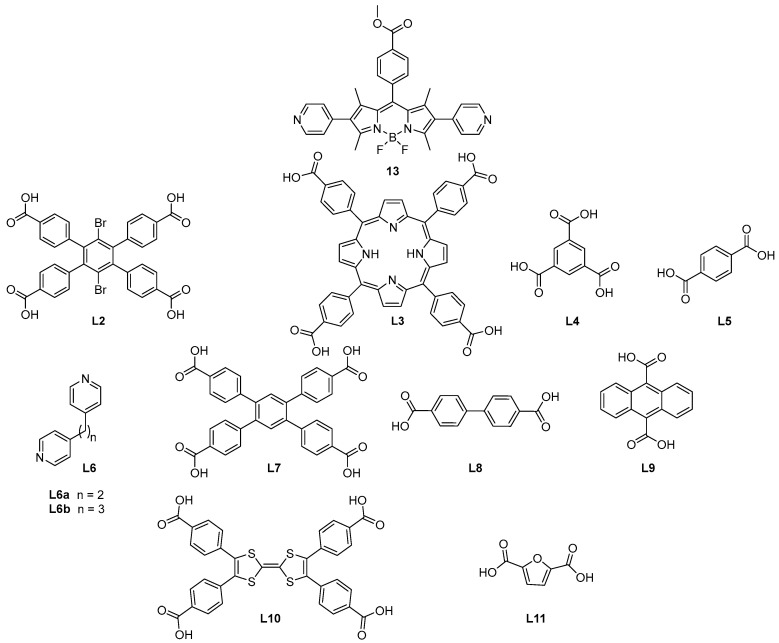
Auxiliary ligands **L2**–**L11** are used along with bidentate BODIPYs for MOF design, as well as bidentate BODIPY **13**.

In this subsection, MOFs containing a coordinating bidentate BODIPY as well as a second non-BODIPY auxiliary ligand will be discussed. The resulting materials are supposed to contain the BODIPY and the auxiliary ligand in proportions defined by the compound’s stoichiometry—if defects are not considered. The BODIPY ligands considered within the studies reviewed in this subsection are essentially the same as for the previous subsection (except **13**), while the auxiliary ligands are of varied structure, denticity, topology, and electronic properties (Scheme 4). The synthesis conditions are summarized in Table 4, as well as other key data. The same remarks as for Section 2.2 apply.

**Figure 6:**
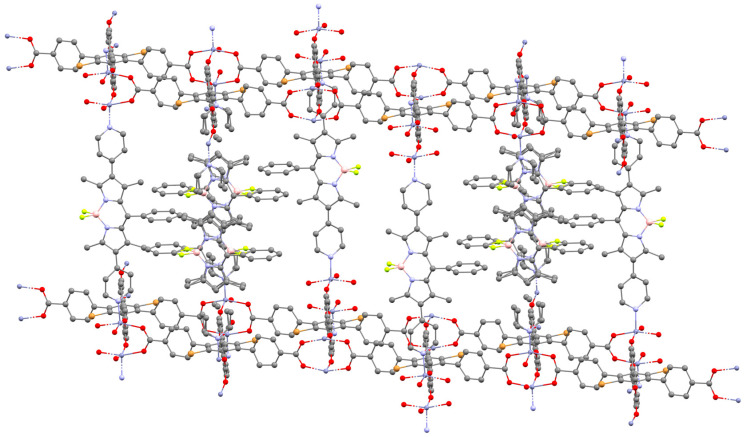
Crystal structure of **1a**@Zn**L2** CCDC number: 860288. Some BODIPYs display disorder. Figure generated with Mercury software [23].

For this type of material, some studies have focused on its **optical properties**. In a foundational example reported in 2011, Farha et al. prepared **1a**, and reacted it with zinc ions, in the presence of two different auxiliary ligands: 4-[2,5-dibromo-3,4,6-tris(4-carboxyphenyl)phenyl] benzoate (**L2**) and tetrakis(4-carboxyphenyl)porphyrin (**L3**): a one-pot method for **L2**, while the porphyrin **L3** was pre-coordinated with zinc before the introduction of **1a** [58]. The BODIPYs are perpendicular to the auxiliary ligands. For the **L2**-based MOF, the BODIPY is excited and emits at 596 nm with an excitation of 520 nm. However, for its **L3** counterpart, the BODIPY performs an energy transfer (FRET) with the porphyrin, which emits at 667 nm. This allows obtaining a material that absorbs a large part of the visible spectrum while emitting a defined wavelength corresponding to the porphyrin. To exemplify a structure typical of MOFs constructed from BODIPYs and auxiliary ligands, the crystal structure of **1a**@Zn**L2** is depicted in Figure 6.

Du et al. reported in 2013 a hybrid material with the same BODIPY **1a** and a metal cluster composed of zinc with either 1,3,5-benzenetricarboxylate (**L4**) or terephthalate (**L5**) as the auxiliary ligand [59]. In both cases, a 3D structure was obtained, featuring two forms of pores, large, interpenetrated openings (361 Å^2^) and small cavities (72 Å^2^). The materials formed with **L4** and **L5** emit in the solid state at 540 nm (λ_exc_ = 512 nm), typical of BODIPY, but the properties were not studied in greater detail.

Hou et al. described in 2015, MOFs with BODIPY **12a**, difunctionalized at the β position with a carboxylic acid function, coordinated to zinc or cadmium clusters, with different pyridine ligands **L6a,b** as auxiliary ligands [60]. The synthesis was carried out in a one-pot method using two equivalents of the auxiliary ligand relative to the fluorophore. The results show that the choice of metal or auxiliary ligand influences the final structure obtained, particularly the configuration of the clusters. In the case of cadmium, the impact of different solvents used during synthesis was also studied, revealing a direct influence on the optical properties of the obtained materials. Indeed, among the five compounds obtained, the maximum emission wavelengths varied from 573 nm to 602 nm (λ_exc_ = 450 nm). Additionally, the quantum yields were determined and differed among the reported examples, though they remained modest (PLQY = 0.67–1.7% for BODIPY@MOF materials, compared to 0.73% for **12a** in the solid state).

Ju et al. recently reported in 2025 three MOFs, two of which incorporated **1a** coordinated to a cadmium node [Cd_2_(COO)_4_] (COO groups belonging to the linkers) [61]. In the first case, the auxiliary ligand is a tetracarboxylated porphyrin **L3** (which is also photoactive), and in the second case, it is 1,2,4,5-tetrakis(4-carboxyphenyl)benzene (**L7**). The choice of the **1a** and **L3** ligands allowed Intrareticular Energy Transfer (IRET), from **L7** to **1a**. This process was very efficient with 88% energy transfer upon 545 nm excitation. By modifying indium tin oxide (ITO) electrodes with the MOFs, photocurrents were determined. IRET allowed photocurrent responses at wavelengths in the 460–550 nm range corresponding to the **1a** absorption. The photoelectrochemical analysis was performed in the presence of sacrificial dopamine (reductant) and O_2_ (oxidant). Using the photoelectrochemical properties of the MOF containing **1a** and **L3**, a biosensor for Vascular Endothelial Growth Factor isoform 165 (VEGF_165_) was designed.

The next studies discussed will focus on **electrochemical applications**. Lei et al. investigated in 2022 the electrochemical behaviour of BODIPY-based MOFs in electrochemiluminescence (ECL)-based sensors. BODIPYs have been reported to be active in ECL (e.g., see ref. [69]). The authors used BODIPY **1a**, coordinated to zinc(II) ions along with terephthalic acid **L5** as an auxiliary ligand [62]. The synthesis was carried out in a one-pot method with one equiv. auxiliary ligand for two equiv. BODIPY. In the final structure, the BODIPYs are placed parallel to each other and perpendicular to the auxiliary ligand, with a **1a**–**1a** distance far greater than in crystals of the pure compound. IR analysis identified the modification of the C=N stretching bands of the pyridine, highlighting the coordination of Zn with N. The distance and orderly arrangement in the MOF could reduce non-radiative deactivation and thus enhance the optical signal. Indeed, after electrochemical reduction, the radical anion form of the MOF was transformed into its excited emissive state after reaction with the sulphate radical anion in a process 24 times more efficient than for an aqueous solution of **1a**. This was exploited in an ECL-based detection method of the biologically important protein telomerase.

Gupta et al. also described in 2023 the use of 1a, this time linked to cobalt(II) with 1,3,5-benzenetricarboxylate (**L4**) as the auxiliary ligand [63]. The obtained solid was characterized by single-crystal X-ray diffraction, revealing alignments of BODIPY perpendicular to **L4**, connected by metal nodes consisting of a single Co^2+^ ion. This material was then incorporated into various carbon composites, enabling the formation of an electrocatalyst for oxygen evolution reactions (OER). OERs are essential for sustainable energy solutions requiring water splitting; however, non-sustainable noble-metal-based catalysts define the state of the art currently. In their study, the authors demonstrated that their MOF outperformed IrO_2_, a benchmark OER catalyst.

In a **PDT** application, Meng et al. reported in 2021 a MOF based on the BODIPY **13** bearing pyridines at the first β position and a 4-(carboxymethyl)phenyl as meso substituent along with Cd^2+^ ions [64]. 4,4′-diphenyldicarboxylate (**L8**) is also present in the structure as an auxiliary ligand. The latter takes a position perpendicular to the BODIPY ligand, resulting in the formula Cd(**L8**)(**13**). The resulting porous material is loaded with the therapeutic agent DOX (doxorubicin, a DNA intercalator used as an anticancer drug). The authors then presented biological results indicating the theranostic potential of their DOX@MOF, as well as the complementarity of dynamic phototherapy (generation of ^1^O_2_ by the BODIPY) with chemotherapy by DOX.

In a 2021 study combining **optical characterization and photocatalysis**, Gupta et al. once again examined **1a**, linked to zinc nodes, with anthracene dicarboxylate (**L9**) as the auxiliary ligand [65]. The synthesis was carried out in a one-pot method with **1a** in a slight deficit relative to the auxiliary ligand. The two different ligands are positioned perpendicular to each other in the unit cell. The authors conducted preliminary photophysical characterization of the MOF. They demonstrated that BODIPY emission is observed when **L9** is excited, and this is correlated with a significant decrease in luminescence lifetime (0.6 ns vs. 12 ns) and the photoluminescence quantum yield of the anthracene fluorophore (0.5% vs. 16%). Subsequently, they also showed that this MOF can be used in the photocatalytic oxidation of DHN (1,5-dihydroxynaphthalene) to juglone (5-hydroxy-1,4-naphthoquinone), a molecule of therapeutic interest.

Finally, studies were focused on **photocatalysis**. In an article dealing with BODIPY **1a**, as well as terephthalate **L5,** Wen, Peng, described a MOF containing a two-zinc node, Zn_2_(COO)_4_ (COO^−^ from terephthalate). The MOF is threefold interpenetrated, and the authors named it CCNU-1. This material was then used in photocatalysis oriented toward the hydrogen evolution reaction. Indeed, a composite material was assembled with platinum(0) nanoparticles. The nanoparticles were synthesized by reduction in H_2_PtCl_6_ in the presence of CCNU-1. The authors provide both electrochemical and photophysical evidence of electron transfer to the Pd nanoparticles. This allowed the authors to report a very high activity of the photocatalyst (4680 µmolg^−1^h^−1^, with an apparent quantum efficiency of 9.06%) [60].

Wen et al. reported in 2024 the integration of BODIPY 1a into two different MOFs, one based on zinc and the other on cadmium. Both MOFs contained the auxiliary ligand 2,5-thiophenedicarboxylate (TDC) (**L10**) [67]. The authors used a one-pot synthesis, introducing two equivalents of H_2_TDC relative to the BODIPY. In the first case, zinc formed a bimetallic node involving four metal–ligand bonds, while cadmium generated a monometallic node with six metal-ligand bonds, resulting in [Zn_2_(**1a**)_2_(**L10**)_2_] and [Cd(**1a**)(**L10**)]∙2H_2_O. For Zn (but not for Cd), an interpenetrated structure was observed. These materials enabled the photocatalytic oxidation of thioanisole to sulfoxides and the hydroxylation of arylboronic acids. In both reactions, the Zn-containing MOF performed better.

Very recently, in 2025, Wen et al. again used the BODIPY **1a** to integrate it into two different MOFs, one based on zinc and the other on cobalt [68]. Both MOFs contain an auxiliary ligand, tetrathiafulvalene-3,4,5,6-tetrakis(4-benzoate) (**L11**). They synthesized these MOFs using a one-pot method, with one equiv. of **L11** for two equiv. of **1a**. Single-crystal. This zinc-based MOF has an interpenetrated structure. The MOFs are used for the photocatalysis of various reactions, such as photoreductive dehalogenation and thiol oxidation. The authors demonstrated that the zinc-based MOF is more effective for reduction, while the cobalt-based MOF is more effective for oxidation.

### 2.4. Tridentate Ligands

**Scheme 5:**
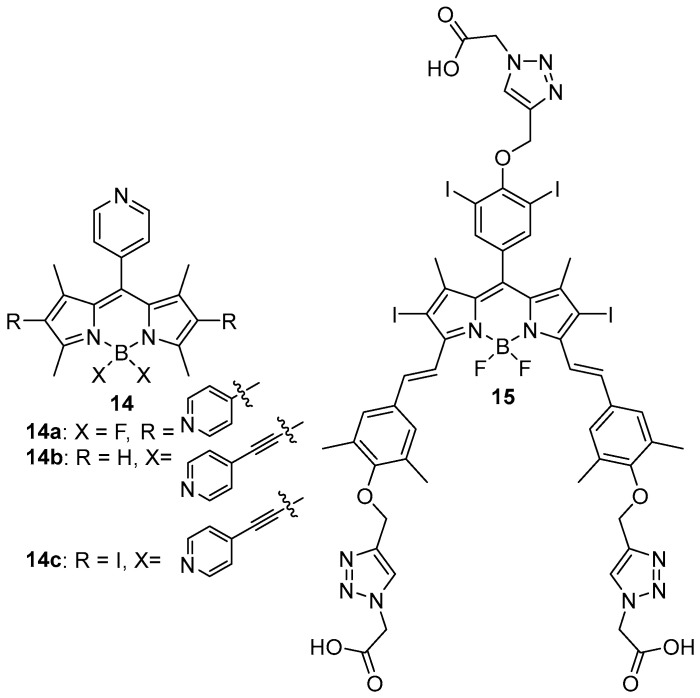
Tridentate BODIPYs **14**, **15**.

To this date, only two publications report the synthesis of MOF containing tridentate BODIPY ligands. The structures of these ligands are depicted in Scheme 5, and the synthesis and characterization details are given in Table 5. The same remarks as for Section 2.2 apply again.

Zang et al., in 2023, described the synthesis of a MOF consisting of silver clusters (Ag_12_(StBu)_6_(CF_3_COO)_6_), connected by three different tridentate BODIPY ligands **14a**–**c** [70]. Ligand **14a** is functionalized with three pyridine groups, one at the meso position and two others at the β positions. Ligand **14b** is also functionalized with a pyridine at the meso position, but the other two pyridines replace the fluorine atoms, with the β positions occupied by hydrogens. Ligand **14c** is similar to 1**4b**, except that the β positions bear iodine atoms. The MOF synthesis was carried out using a one-pot method, followed by slow evaporation to obtain single crystals. These crystals of formula [Ag_12_(StBu)_6_(CF_3_COO)_6_(**14**)_6_]. This synthesized material is capable of capturing pollutant gases, such as sulphur mustard, and allows their **photocatalytic decontamination**.

Guo et al., in 2023, formed a MOF from zirconium clusters (Zr_6_O_4_(OH)_4_) incorporating BODIPY **15** [71]. This peculiar BODIPY is functionalized with three similar long arms at the meso and α positions, each featuring triazole groups formed after a copper-catalyzed azide-alkyne cycloaddition (CuAAC, flagship reaction of Click Chemistry), resulting in a coordinating carboxylate group at the end of each arm. The β positions bear iodine atoms. TEM-EDX studies allowed for elemental mapping of the atoms present and their distribution within the structure, revealing good homogeneity. However, the sample appeared to have low crystallinity, as SEM observations showed particles in the form of small beads, and powder X-ray diffractograms indicated moderate crystallinity. The material displayed potential as an **anticancer agent**, as it has been demonstrated to selectively accumulate in 4T1 tumour cells while exhibiting good stability.

## 3. BODIPYs Attached to MOFs by Organic Reactions

In this section, a limited number of examples will be discussed. These are conceptually designed by appending BODIPY moieties to entities known to behave as linkers between metal nodes, as opposed to BODIPY-centred ligands (see Section 2.2, Section 2.3 and Section 2.4). Hence, the structures of the resulting BODIPY@MOF materials are expected to be very similar to those of the parent MOFs.

**Scheme 6:**
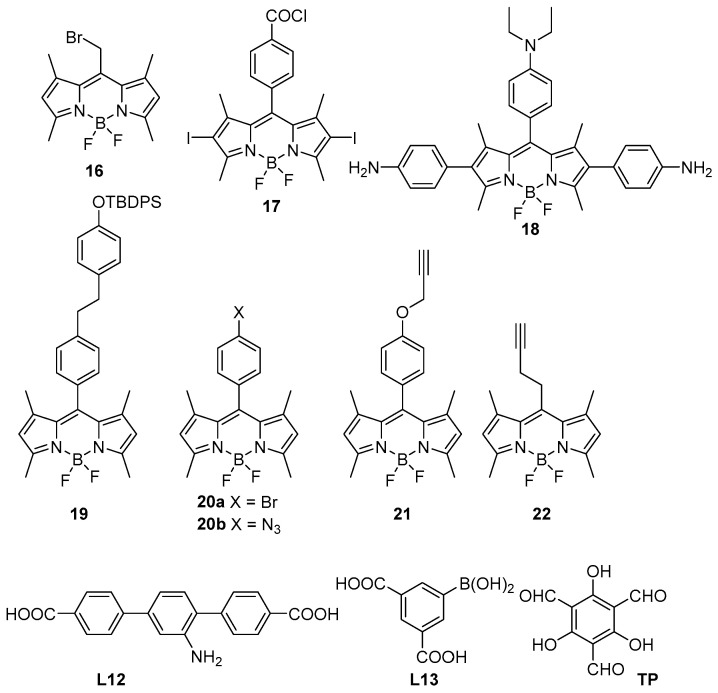
BODIPYs **16**–**22** used in the covalent functionalization of MOFs, as well as auxiliary ligands **L12**, **L13**, and **TP**, reactant.

To obtain the target materials, only post-functionalization strategies were developed, which allow divergent functionalization from a single MOF precursor. However, they face challenges typical of heterogeneous reactions, and the MOF structure must be compatible with the synthetic conditions envisioned. The BODIPYs used for these strategies are summarized in Scheme 6, and the synthesis conditions and quantitative characterization of the BODIPY loading are summarized in Table 6. The synthetic conditions are typically milder (often RT) than methods requiring the integration of BODIPY-centred ligands (Section 2.2, Section 2.3 and Section 2.4), with the notable exception of the last entry in which harsher conditions are required due to the concomitant formation of MOF with a Click reaction. Due to the limited number of examples, articles in this section are presented chronologically rather than by application.

In an early (2009) and seminal example, Lin and co-workers reported the postsynthetic functionalization of a MIL-101(Fe) nanosized MOF described by the formula Fe_3_(µ_3_-O)Cl(H_2_O)_2_(L)_3_ (where L = terephthalate or 2-aminoterephthalate) [72]. The incorporation of up to 17.5 mol-% of 2-aminoterephthalate did not result in changes detectable by PXRD. Then, reaction with brominated BODIPY **16** (about the same mass as the MOF) allowed the covalent grafting of the BODIPY on the structure. The **16** loading was quantified by digesting the MOF (Na_4_EDTA solution) and assaying the **16** released in solution by UV-Vis spectrophotometry. The authors stress that their **16**@MOF material is nonemissive due to quenching by d-d transitions of Fe^III^. Their BODIPY-loaded nanoparticles of their MOF were found to be unstable in the common PBS buffer (half-life t_1/2_ = 2.5 h, for 8 mM buffer at 37 °C) by assaying the release of **16**. The t_1/2_ could be raised to 16 h by coating the particles with silica. The silica-coated particles proved efficient in delivering the BODIPY dye to HT-29 cancerous cells. Their article also included the application of the same functionalization strategy for an anticancer drug.

In 2016, Li, Zhao, Wang, and co-workers described the post-modification of a Zr^IV^ MOF with a diiodo-BODIPY **17**. The zirconium MOF was UiO-68-NH_2_, which was obtained from aminotriphenyldicarboxylic acid (**L12**). Hence, a smooth reaction allowed the coupling of the acyl chloride of **17** to the amine group of **L12** within a preformed MOF [73]. The as-synthesized UiO-68-NH_2_ was treated with 10% triethylamine in ethanol to deprotonate the amine groups. Then, the solvent mixture was reacted with **17**. The loading of BODIPY was determined by X-ray fluorescence using the heavy elements Zr (from the MOF) and I (from **17**), indicating that up to 11% of the organic ligands in UiO-68-NH_2_ were functionalized with **17**. Then, the authors verified that the photocatalytic activity of **17** towards the dehydrogenative cross-coupling of *N*-phenyl-1,2,3,4-tetrahydroisoquinoline with nitromethane under green LED irradiation was maintained when **17** was immobilized within the MOF. Other oxidative reactions were also successfully tested.

Chen, Wang, Xie et al. have reported in 2020 an original strategy to post-functionalize UiO-66-NH_2_ [74]. The preformed MOF was exposed to a diamine-functionalized BODIPY **18** along with 1,3,5-triformylphloroglucinol (**TP**). Through the formation of imine bonds, the BODIPY dye was covalently immobilized in the MOF. PXRD showed that the crystallinity was maintained in the process. The solid material acquired the characteristic colour of the BODIPY dye. UV-Vis, IR, and NMR spectroscopies proved the immobilization of BODIPY in the MOF. TGA analyses showed that the added organic part consists of around 15 wt-%. The presence of the diethylamino moiety in **18** allowed the achievement of smart properties in the biological context (photodynamic therapy) for the final MOF. Indeed, the final MOF was active in type I photodynamic therapy, with an extra activity against HeLa cancerous cells versus normal L929 cells. This selectivity stems from the protonation of the diethylamino group. If the latter is protonated, the final MOF releases hydroxyl radicals. If not, there is a competitive PET process implying -NEt_2_.

Fan, Yan, Ke, and co-workers described in 2022 a MOF based on Eu^3+^ and the ligand BBDC (**L13**) doped with the BODIPY **19** [75]. According to these authors, the covalent immobilization strategy consisted of the formation of an adduct with bridging fluoride between the **19** and the boronic acid group of **L13** within the MOF. The inclusion of up to 9.5 wt-% of **19** with respect to BBDC was performed in a one-pot approach in solvothermal conditions. The inclusion of **19** resulted in lower crystallinity (PXRD). The inclusion of growing amounts of **19** changed the morphology of the material from nanorods to nanosheets. From the fluorescence spectroscopy, elemental mapping showed a uniform distribution of elements (including Si, only present in **19**), which supports the formation of an adduct, although the loading of **19** in the final material was not quantified. The material with an initial loading of 4.8 wt-% was investigated for applications. Eu^3+^ is inherently emissive (l_max_ = 616 nm), and for the final material, a band appeared in the emission spectrum at 420 nm for the adduct between **19** and the boronic acid group of BBDC. This allowed us to design ratiometric detection approaches for F^−^ and H_2_O_2_ based on the reactivity of those analytes with boronic acid moieties.

Li and co-workers described in 2022 a peculiar MOF with a thiazolothiazole-based dicarboxylate linker, isoreticular to the terphenyl-based UiO-68 (ZrTc). The thiazole nitrogen could be alkylated post-synthetically. The resulting alkylated MOF had improved NLO properties, displaying fluorescence after efficient multi-photon excitation in the NIR-II region [78]. In 2025, they reported the propargylation of the thiazole in a preformed ZrTc MOF, followed reportedly by a copper-catalyzed azide-alkyne cycloaddition (reaction with a BODIPY carrying a para-azidophenyl substituent in the meso position) [76]. Examination of the analytical data reported for the azido-BODIPY casts a doubt on this result, as no IR band associated with N_3_ was observed in the BODIPY starting material **20a**, whose precursor **20b** displayed a para-bromophenyl group. In our opinion, it is likely that encapsulation of **20b** was performed in the ZrTc MOF rather than click CuAAC of its azidated counterpart **20a**. After loading the BODIPY, the MOF nanoparticles were coated with hyaluronic acid. On these final materials, named ZTBH, the authors performed extensive photophysical and biological experiments showing that efficient two-photon photosensitization was achieved under NIR laser irradiation. Moreover, fluorescence lifetime measurements were indicative of changes in cellular microenvironments during PDT.

Guo, Liu, Zhang et al. described in 2024 a new MOF following overall the UiO architecture but with a Co_16_ cluster containing both azide and formate ions [77]. These authors reported an impressive array of MOFs by varying the ligands bridging the Co_16_ clusters. Among those was the usual terephthalate. The Co_16_-MOF with terephthalate was coupled by azide–alkyne cycloaddition with BODIPYs **21** and **22**. Hence, the BODIPY was directly attached to the Co_16_ clusters. Control experiments proved the covalent attachment, and the presence of significant amounts of copper contaminants (CuAAC catalyst) was ruled out by ICP. The BET surface area was also reduced from 194 m^2^g^−1^ to 155 m^2^g^−1^ upon covalent grafting. In the photocatalytic oxidative coupling of benzylamine, the authors then demonstrated that the clicked **21**-Co**_16_**-MOF clearly outperformed the parent material. The clicked MOF achieved nearly quantitative yield, which was attributed to a synergistic behaviour between the metal cluster and the BODIPY. Notably, the BODIPY allowed the photogeneration of the superoxide ion, which was not possible with the pristine material.

## 4. Non-Coordinating BODIPYs

**Scheme 7:**
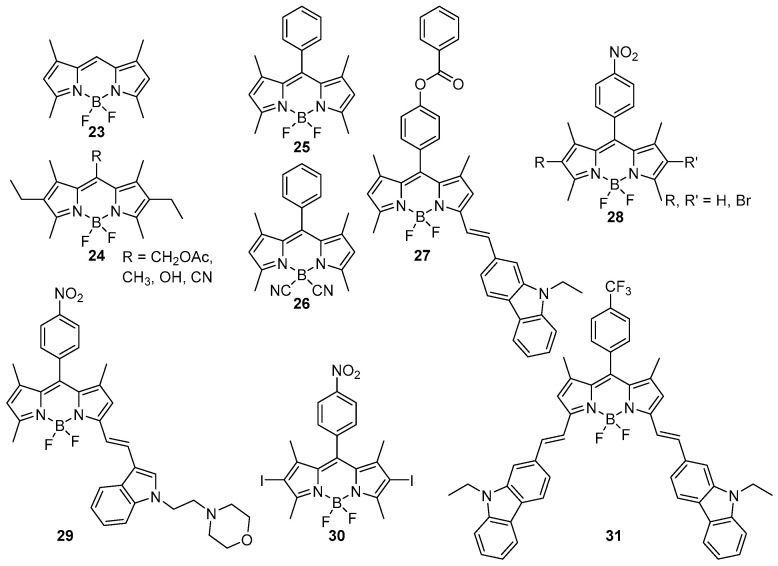
Non-coordinating BODIPYs **23**–**31**.

In this section, examples of hybrid BODIPY@MOF materials *not* relying on the coordination of the BODIPY on metal nodes will be reviewed. To this end, the BODIPYs used lack coordinating moieties, freeing several positions for other functionalities (Scheme 7). The main practical advantage of this strategy is the operational simplicity, as shown by the synthesis conditions outlined in Table 7. However, for this strategy, there is the obvious possibility of BODIPY guest leakage from the MOF host. Indeed, a simple tetramethylated BODIPY **23** was used as a probe to investigate the transport process in solvent-filled NU-1008 crystallites. NU-1008 has large 1D hexagonal channels [79]. The impact of post-synthetic modifications of the latter MOF on the transport properties was investigated [80]. This proof-of-concept study paves the way for a deeper understanding of mass transport phenomena in MOFs, the control of which is important for numerous applications, but also underlines the special considerations that must be made to avoid leakage of the BODIPY from the MOF when the hybrid is exposed to solvents. In this section, several examples of materials arising from non-covalent interactions between a BODIPY and an MOF scaffold will be discussed in detail.

**Luminescent BODIPY@MOF materials** not relying on coordination interaction were studied in 2018 by Friščić, Cosa, and co-workers with BODIPY dyes of the **24**-type encapsulated in ZIF-8 [81]. The rationale of this study was the very good size matching between the pore diameter and the BODIPY dimensions in combination with smaller pore apertures, which could lead to a stable hybrid material. Most of the study was performed with a BODIPY bearing a methyleneacetoxy group in the meso position, with other substituents introduced to further exemplify the method. Synthetically, solvent-free mechanochemical (ion- and liquid-assisted grinding, ILAG [88]) or accelerated ageing (AA) [89] approaches were used in the presence of 0.05 to 5 wt-% of BODIPY. The encapsulation could be appreciated by the colour of the samples under visible light, along with a distinct luminescence upon UV irradiation. UV assays of solutions obtained by thoroughly washing the samples (MeOH, DCM) demonstrated a nearly total for and very good encapsulation for ILAG and AA approaches, respectively. PXRD and N_2_ sorption experiments yielded very similar results to pristine ZIF-8, as expected from low dye loading. Fluorescence quantum yields were compared to the solution measurement (67%) and ranged from 68% for the lowest loaded sample, while the quantum yields dropped to 7.8% for the highest loading. The lowest loading solid-state sample also displayed a luminescence lifetime quite similar to the value reported for the solution (6.76 vs. 7.5 ns), highlighting a monomeric emission in the diluted solid state. This, along with the observation of bathochromic shifts in the emission spectra and shortened emission lifetimes, induced the authors to postulate that weakly emissive H-aggregates could be formed with increased dye loading, and that these could act as trap sites in energy transfer processes with molecules in neighbouring pores. Very interestingly, the authors demonstrated that the incarceration in ZIF-8 improved dramatically the photostability of the BODIPY guest.

In a very recent article (published only a few days after the submission of this review), Janick et al. studied the encapsulation of BODIPYs **25** (37% PLQY, t = 5.7 ns in CHCl_3_) and **26** (89% PLQY, t = 4.8 ns in CHCl_3_) into four different MOFs, UiO-66, MOF-808, DUT-67 and MIP-206 [82]. At first, they focused on the case of UiO-66, but with **25,** they did not obtain any positive result, likely due to the acidity of the defective UiO-66 structure. On the other hand, **26** is more stable towards acidic conditions and was successfully used in two strategies. The first one is a post-functionalization approach. For the post-synthetic method, they incorporated 0.44 wt-% of **26**, associated with a sharp decrease in the BET surface area. The second integration approach is the one-pot method; for this, they introduced different quantities in the synthesis between 3 and 15 mmol/L. This resulted in up to 7.2 wt-% BODIPY in their MOF. The study of the BET surface area shows the same phenomenon as the post-synthetic approach. In terms of luminescence quantum yields, the best sample of the one-pot approach is **26**@UiO-66_1.1wt%_ with 32% (t = 5.1 ns), compared with the post-synthetic approach sample, with **26**@UiO-66_0.44wt%_ and a quantum yield of 29% (t = 5.1 ns). Subsequently, they performed the same studies with MOF-808, DUT-67, and MIP-206. For the MOF-808, they obtained the best sample with a post-synthetic approach, incorporated 0.57 wt-% with a quantum yield of 48% (t = 6.5 ns). For DUT-67, they obtained the best sample with the post-synthetic method, also, with 0.51 wt-% incorporated and a quantum yield of 77% (t = 6.8 ns). Finally, for MIP-206, they only performed the post-synthetic approach and obtained a sample with 0.3 wt-% and a quantum yield of 47% (t = 7.9 ns). The results highlight quantum yields and luminescence lifetimes similar to or higher than those in the solid state as compared to solution, which is compatible with the MOF, allowing a “solid solution” environment for efficient BODIPY luminescence.

**Sensor applications** were reported in several publications. Shen, Zhang, Huang, and co-workers reported in 2021 the use of **27**@ZIF-8 to detect carboxylesterase 1 (CES1) [83]; **27** was used again in a very small amount compared to 2-methylimidazole (less than 0.3 mol-%). The authors used UV-Vis spectroscopy to assay the integration of the BODIPY **27** in their MOF (assay of a digested sample in DMSO/citrate buffer) and demonstrated 76% integration. They used their probe to detect CES1 by a decrease in the fluorescence intensity (initial quantum yield: 65% as deduced from indirect measurements) and reported a limit of detection (LOD) of 1.15 ng/L. This allowed the visual monitoring of CES1 activity in living cells. Moreover, the pesticide chlorpyrifos inhibits CES1, allowing an indirect assay of this pesticide. Indeed, a linear curve was obtained by comparing the luminescence intensity of the samples in the absence and presence of increasing concentrations of the pesticide after a fixed incubation time.

Xue, Chen et al. reported in 2023 [84], the use of a copper-based terpyridine MOF, initially published by Konar and co-workers [90], to host BODIPY molecules of the **28**-type. The MOF was prepared in the absence of **28** molecules, which were subsequently loaded in solution. Finally, PEG-600 was added as a stabilizer. In the same work, non-MOF nanoparticles were also used (porous silica and polystyrene-co-acrylic acid nanoparticles). The different materials were considered in an applied study towards the colorimetric detection of volatile organic compounds in the solid state. While the application was thoroughly studied, the characterization of the MOFs themselves was rather limited. In particular, the authors did not quantitatively investigate the loading of non-coordinating BODIPYs of type **28** in their MOFs.

In another article based on a similar strategy, He, Shen et al. reported in 2022 ZIF-8 loaded with BODIPY **29**, to design a red-emitting nanocomposite for the detection of nitroreductase (NTR) in living cells [85]. NTR is correlated with hypoxia, which is typical of solid tumours. Their synthetic strategy relied on the addition of a small amount (**29** as an additive in the synthesis of ZIF-8, followed by ethanol washings in the synthesis work-up. Direct evidence of the encapsulation of **29** was provided by XPS and microscopy element mapping; however, the BODIPY contents in the final material were not quantified. The authors demonstrated the use of **29**@ZIF-8 as a sensor for NTR, with a fluorescence enhancement upon exposure of the material to the target enzyme, both with isolated enzymes and full cells.

In addition to these works, a report by Zhang et al. mentions MOF and BODIPYs in more sophisticated assembly with a polymer as a third party. In this work, nanoprobes relying on FRET between a luminescent MOF and a C, O-BODIPY appended to a polymeric chain were designed for pH and temperature sensing based on modulation of donor(MOF)-acceptor(BODIPY) distance [91].

**Photodynamic therapy** (PDT) was reported in 2018 by Li, Dong, and co-workers using diiodo-BODIPY **30** [86]. The presence of iodine atoms increases the quantum yield for the generation of ^1^O_2_, with applications in PDT. The authors chose ZIF-90 as host and underline the compatibility of the dimensions of molecule **30** (10.8 × 10.4 Å) with the pore diameter of the MOF (11.2 Å). Based on ICP measurements (no experimental details were provided), they claimed a loading of 25.4 wt-%, which is near the saturation of ZIF-90s pores. PXRD measurements showed that the ZIF structure was not disrupted, while the BODIPY guest was not detected by ^13^C solid-state NMR and IR. Such a high weight percentage seems inconsistent with the IR and NMR results. Despite this, the **30**@ZIF-90 composite exhibited impressive biological properties, benefiting from a synergy between the MOF host and the BODIPY guest. Inclusion in the MOF greatly improved the photostability of BODIPY **30**. Moreover, ZIF-90 is stable at physiological pH (blood circulation, pH = 7.4) but less so at slightly acidic pH values (pH of certain cellular compartments), due to imidazolate protonation. Biologically, the resulting positive surface charge of the **30**@ZIF-90 nanoparticles enhances membrane crossing and selective uptake in cancer cells (with microenvironments at pH = 6.5) and also induces mitochondrial targeting. Finally, the authors demonstrated the potential of their material in PDT (high-light-induced vs. low dark cytotoxicity). Overall, their strategy was complementary to purely molecular PDT approaches, with fewer challenges in organic synthesis.

In 2022, Shen, Luan et al., reported the encapsulation in ZIF-8 of an extended conjugated BODIPY **31**, with the objective of designing efficient materials for PDT [87]. **31** was chosen among a set of BODIPY photosensitizers and proved to be more efficient as compared to compounds bearing only one carbazole moiety. For **31**@ZIF-8, the synthetic approach used again a very small amount of **31** as an additive in the synthesis of ZIF-8. The authors characterized the hybrids by several techniques, including microscopy, DLS, XPS and EDX element mapping, showing the formation of well-defined 250 nm particles with uniform distribution of B and F elements, hinting at a good encapsulation of **31**. Interestingly, a MOF/BODIPY synergy was envisioned by the authors, with ZIF-8 able to promote the dismutation of H_2_O_2_ and thus increase the concentration of O_2_. This is of importance considering that tumours are typically hypoxic, thus reducing the efficiency of PDT based on the generation of singlet dioxygen. This effect was proven in vitro, and biological tests performed on living mice affected by tumours suggested a superior efficiency of the **31**@ZIF-8 hybrid as compared to molecular **31**.

## 5. Conclusions and Outlook

In this review article, we sought to summarize a recent research area building on the impressive properties of BODIPYs in their molecular form in solution, as well as the unique characteristics of MOF among the palette of hybrid materials. The examples discussed within this review provide a variety of ideas useful for future works, both for the synthetic conditions, materials characterization and applications. Current limitations and shortcomings of the literature differ following the integration strategy. On the one hand, for stoichiometrically defined materials with BODIPY-centred ligands, the main challenge is to ensure the compatibility of the synthetic conditions of MOF (often high temperature, and in the presence of strong Brønsted and Lewis Acids) with the BODIPY. On the other hand, for all other strategies, the crucial aspects of quantification of the BODIPY loading and verification of the MOF integrity arise. In the future, we anticipate discoveries in all of the research axes outlined in the paper, BODIPY-derived ligands as linkers, covalent grafting, and encapsulation. We believe that coordination chemistry of covalent-grafting procedures, though more challenging, offers greater opportunities in terms of fine-tuning of the hybrid materials as compared to encapsulation approaches, which might be, on the other hand, simpler in practice. The use of advanced and *quantitative* characterization techniques seems of paramount importance, especially when BODIPY is used as a dopant and not a stoichiometric linker of the MOF. The state of the art in this area is currently under definition, as the characterization efforts of the BODIPY-containing materials are quite heterogeneous in the reviewed articles.

This research area is quite new but promises further developments. The examples known to date demonstrate that the integration of BODIPYs within MOFs presents a fertile ground for discovery, innovation, and applications. The synergistic combination of BODIPYs’ photophysical and/or photochemical properties with the structural versatility and porosity of MOFs opens up avenues for applications in fields such as luminescent or light-harvesting materials, photodynamic therapy, sensing, and photocatalysis, which were only partly explored in recent years. The potential for new breakthroughs in this area is quite vast, and continued interdisciplinary collaboration will be key to unlocking new capabilities of BODIPY@MOF systems.

## Figures and Tables

**Table 1:** Synthetic conditions of the MOF-5, UiO-66, and ZIF-8.

MOF	Formula	General Synthetic Method	Reaction Medium	Temperature (°C)	Time (h)	Reference
MOF-5	Zn_4_O(C_8_H_6_O_4_)_3_	Slow vapour diffusion	Et_3_N/ Toluene/DMF	RT	NR ^1^	[28]
UiO-66	Zr_6_O_4_(OH)_4_(C_8_H_4_O_4_)_6_	Solvothermal	DMF	120	24	[20]
ZIF-8	Zn(C_4_H_6_N_2_)_2_		DMF	140	24	[21]

^1^ Not reported in the original article, but vapour diffusion usually requires several days.

**Table 2:** Overview of synthetic conditions, quantification of BODIPY, and applications for MOFs containing monodentate BODIPYs.

Ligand, MOF	Approach (Synthetic Conditions)	Quantification of BODIPY in the MOF	Application	Reference
**2**, UiO-66	SALE (DMF 65 °C, 24 h)	27.9% (ICP-MS, UV-Vis)	PDT	[43]
**3**, PCN-222	SALI (MeCN/DMSO 3:2, 60 °C, 24 h)	44% relative to the porphyrin ligand of PCN-222 (Liquid NMR after decomposition)	PDT	[44]
**4**, ZIF-90	One-pot (H_2_O, RT, overnight)	ND (0.3 wt-% relative to imidazole ligand used)	Detection of analytes	[45]
**5**, ZIF-8	One-pot (DMF, RT, 12 h addition of BODIPY delayed by 2 h)	ND (2.5 mol-% relative to the imidazole ligand used)	Antibacterial agent	[46]
**6**, NU-1000	SALI (MeCN, 60°C, 24 h)	0.75/Zr_6_ node (X-ray fluorescence)	Photocatalysis (detoxification of sulphur mustard)	[47]
**3** or **4,** nPCN-222	SALI (MeCN/DMSO 3:2, 60 °C, 24 h)	3.5–3.6/Zr_6_ node (Liquid NMR after decomposition)	Light harvesting/FRET	[48]
**6** or **7**, MOF-5	One-pot (DMF/NEt_3_, RT, 2.5 h)	Up to 17.7 wt-% (Spectrophotometric assay of washings)	Luminescent materials	[49]

**Table 3:** Overview of synthetic conditions, MOF characterization, and applications for MOFs containing bidentate BODIPYs.

Ligand, Cluster	Approach (Synthetic Conditions)	Proof of Structure	Application	Reference(s)
**1a**/Cu_4_I_4_ **1b**/Cu_2_I_2_	One-pot (MeCN, 55 °C, 24 h)	SCXRD compared with PXRD	Luminescent materials ^1^	[14]
**8**/RE ions ^2^	One-pot (DMF/Acetate/Water, 60 °C, 24 h)	SCXRD compared with PXRD	PDT	[51,52]
**9**/Zr_6_O_8_	SALE (DMF, 70 °C, 96 h) ^3^	SCXRD compared with PXRD before and after SALE ^1^H NMR (100% of exchange)	PDT	[53]
**10**/Cu_3_L_3_	One-pot (nBu-OH/BnOH, 120 °C, 72 h)	PXRD compared with the predicted model ^4^	Photocatalysis (Organic synthesis)	[54]
**11**/Zr_6_O_8_ **11**/Zr_12_O_22_	One-pot (Formic acid, 70 °C, 72 h)	Elemental analysis SCXRD compared with PXRD	Photocatalysis (CO_2_ reduction)	[55]
**12**/Zr_6_O_8_	One-pot (DMF, 120 °C, 24 h)	Elemental analysis SCXRD compared with PXRD	Photocatalysis (Organic synthesis)	[56]

^1^ Attempted application; however, the authors described the quenching of luminescence in the solid state. ^2^ Trivalent rare earth ions. ^3^ **9** in large excess: 100 mg/30 mg of MOF. ^4^ Albeit with limited crystallinity.

**Table 4:** Overview of synthetic conditions, MOF description and applications for MOFs containing bidentate BODIPYs.

Ligand/Metal/ Aux. Ligand	Synthetic Conditions ^1^	Proof of Structure	Application	Reference
**1a**/Zn^2+^/**L2** or **L3**	DMF, 80 °C, 2 h addition of BODIPY delayed by 20 h with HNO_3_/EtOH	SCXRD compared with PXRD	FRET	[58]
**1a**/Zn^2+^/**L4** or **L3**	DMF, HNO_3_/EtOH, 80 °C, 48 h	SCXRD compared with PXRD	Luminescent Materials	[59]
**12a**/Zn^2+^ or Cd^2+^/**L6**	DMF, HNO_3_/EtOH, 80 °C, 48 h	SCXRD compared with PXRD	Luminescent Materials	[60]
**1a**/Cd^2+^/**L3**	DMA/HNO_3_, 90 °C, 72 h	SCXRD compared with PXRD	Detection of analytes	[61]
**1a**/Zn^2+^/**L5**	DMF, HNO_3_/EtOH, 85 °C, 48 h	SCXRD compared with PXRD	Detection of analytes	[62]
**1a**/Co^2+^/**L4**	DMF/water, 85 °C, 72 h ^2^	SCXRD compared with PXRD	Electrocatalysis (OER catalyst)	[63]
**13**/Cd^2+^/**L8**	DMF/EtOH, HNO_3_, 80 °C, 72 h	SCXRD compared with PXRD	PDT	[64]
**1a**/Zn^2+^/**L9**	DMF/EtOH, HNO_3_, 90 °C, 18 h	SCXRD compared with PXRD	Photocatalysis (Organic synthesis)	[65]
**1a**/Zn^2+^/**L5**	DMA/CH_3_OH, HNO_3_, 90 °C, 48 h	SCXRD compared with PXRD	Photocatalysis (Hydrogen evolution reaction)	[66]
**1a**/Zn^2+^ or Cd^2+^/**L10**	DMA/EtOH, HNO_3_, 90 °C, 24 h	SCXRD compared with PXRD	Photocatalysis (Organic synthesis)	[67]
**1a**/Zn^2+^ or Co^2+^/**L11**	DMF/MeOH, 70 °C, 48 h	SCXRD compared with PXRD	Photocatalysis (Organic synthesis)	[68]

^1^ All these stoichiometric reactions were performed by introducing the BODIPY and the auxiliary ligand simultaneously. ^2^ Auxiliary ligand: BODIPY ratio: 2 to 3.

**Table 5:** Overview of synthetic conditions, MOF description, and applications for MOFs containing tridentate BODIPYs.

Ligand/ Cluster	Synthetic Conditions	Proof of Structure	Application	Reference
**14**/Ag_12_(SR)_6_(CF_3_COO)_6_ R = *^t^*Bu, *^i^*Pr	Ethanol/Acetonitrile (1:1), PhPO_3_H_2_ (2–5 d)	SCXRD compared with PXRD	Photocatalysis (decontamination)	[70]
**15**/Zr_6_O_8_	DMF, RT, 6 h	SCXRD compared with PXRD	PDT	[71]

**Table 6:** Overview of synthetic conditions, quantification of BODIPY, and applications for MOFs covalently functionalized with BODIPYs.

BODIPY, MOF	Reaction (Synthetic Condition)	Quantification of BODIPY in the MOF	Application	Reference
**16**, MIL-101(Fe)	Nucleophilic Substitution (excess BODIPY, THF, RT, 2 days)	~10% (UV-Vis)	Biologically targeted dye delivery	[72]
**17**, UiO-68-NH_2_	Amine acylation (THF, RT, 1 day)	11% of the organic ligands functionalized (XRF)	Synthetic photocatalysis	[73]
**18**, UiO-66-NH_2_	Imine formation (DMF, TFA, **TP**, RT, overnight)	15% wt-% (TGA, total added organic content ^1^)	PDT	[74]
**19**, Eu^III^-**L13** MOF	Adduct formation on the boronic acid ^2^ (DMF/Ethylene glycol 7:3, autoclave, 120°)	ND (up to 9.5 wt-% loading)	Detection of analytes	[75]
**20**, ZrTc ^3^	Azide-alkyne cycloaddition ^4^	ND	PDT and biological imaging	[76]
**21** or **22,** Co_16_ MOF ^5^	Azide-alkyne cycloaddition ^2^ (DMF/MeOH 1:1, autoclave, 140 °C, cat.: CuSO_4_/ascorbic acid)	ND (proof of grafting by control experiments)	Synthetic photocatalysis	[77]

^1^ Added organic content from imine formation with TP and BODIPY **18**. ^2^ One-pot reaction with MOF synthesis. ^3^ MOF isoreticular to UiO-68. ^4^ See text below. ^5^ Architecture similar to the UiO series, cobalt clusters bearing azide ions, reaction performed during MOF synthesis.

**Table 7:** Overview of synthetic conditions, quantification of BODIPY and applications for MOFs encapsulating non-coordinating BODIPYs.

BODIPY, MOF	Approach (Conditions)	Quantification of BODIPY in the MOF	Application	Reference
**24**, ZIF-8	Mechanochemistry ^1^	Up to 100% encapsulation of up to 5 wt-% BODIPY (UV-Vis assay of washings)	Luminescent Materials	[81]
**26,** UiO-66, MOF-808, DUT-67 and MIP-206	Post-synthetic soaking of the MOF in a DCM solution of the BODIPY (RT, 5 days) OR One-pot (DMF, 80 °C, 12 h)	UV-vis assay after MOF digestion in KOH.Post-synthetic: 0.3–0.57 wt-%One-pot 1.9–7.2 wt-%	Luminescent Materials	[82]
**27**, ZIF-8	One-pot encapsulation during MOF synthesis (Water, RT, 12 h)	76% integration of 0.3 wt-% BODIPY (UV-Vis assay of washings)	Detection of analytes	[83]
**28**, copper-based terpyridine MOF	Post-synthetic soaking of the MOF in DMF solution of BODIPY	ND (1% wt-% of BODIPY used)	Detection of analytes	[84]
**29**, ZIF-8	One-pot encapsulation during MOF synthesis (Water, RT, overnight)	ND (<0.15 mol% of BODIPY used)	Detection of analytes	[85]
**30**, ZIF-90	One-pot encapsulation during MOF synthesis (DMF, trioctylamine, <0 °C, 1 min)	25.4 wt-% (ICP) ^2^	PDT	[86]
**31**, ZIF-8	One-pot encapsulation during MOF synthesis (Water, RT, 12 h)	ND (0.2 wt-% of BODIPY used)	PDT	[87]

^1^ See text for more details. ^2^ No details given, see text.

## Data Availability

No new data were created.

## Abbreviations

The following abbreviations are used in this manuscript:

AA	Accelerated Ageing
ACQ	Aggregation-Caused Quenching
AIE	Aggregation-Induced Emission
BODIPY	Boron-Dipyrromethene
CCDC	Cambridge Crystallographic Data Centre
CES1	Carboxylesterase 1
CuAAC	Copper-catalyzed Azide-Alkyne Cycloaddition
DOX	Doxorubicin
ECL	Electrochemiluminescence
EDX	Energy-Dispersive X-ray Spectroscopy
FRET	Förster Resonance Energy Transfer
ICP-MS	Inductively Coupled Plasma Mass Spectrometry
ILAG	Ion-and Liquid-Assisted Grinding
IR	Infrared Spectroscopy
IRET	Intrareticular Energy Transfer
ITO	Indium Tin Oxide
LED	Light-Emitting Diode
MIL	*Matériaux de l’Institut Lavoisier*
MOF	Metal–Organic Frameworks
NIR	Near-Infrared
NMR	Nuclear Magnetic Resonance
NLO	Nonlinear Optics
NTR	Nitroreductase
NU	Northwestern University
OER	Oxygen Evolution Reactions
PCN	Porous Coordination Networks
PDT	Photodynamic Therapy
PET	Photoinduced Electron Transfer
PXRD	Powder X-ray Diffraction
ROS	Reactive Oxygen Species
SALE	Solvent-Assisted Ligand Exchange
SALI	Solvent-Assisted Ligand Incorporation
SCXRD	Single-Crystal X-ray Diffraction
SEM	Scanning Electron Microscopy
SPDT	Sonophotodynamic Therapy
TEM	Transmission Electron Microscopy
TGA	Thermogravimetric Analysis
TDC	2,5-thiophenedicarboxylate
UiO	University of Oslo
UV-vis	Ultraviolet-Visible Spectroscopy
VEGF	Vascular Endothelial Growth Factor
XPS	X-ray Photoelectron Spectroscopy
ZIF	Zeolitic Imidazolate Framework
